# Strain-dependent differences in corticolimbic processing of aversive or rewarding stimuli

**DOI:** 10.3389/fnsys.2014.00207

**Published:** 2015-02-04

**Authors:** Diego Andolina, Stefano Puglisi-Allegra, Rossella Ventura

**Affiliations:** ^1^Dipartimento di Scienze Cliniche Applicate e Biotecnologie, Università degli Studi dell’AquilaL’Aquila, Italy; ^2^Santa Lucia FoundationRome, Italy; ^3^Dipartimento di Psicologia and Centro ‘Daniel Bovet’, Sapienza Università di RomaRome, Italy

**Keywords:** strain, neurotransmission, corticolimbic, rewarding stimuli, stress

## Abstract

Aberrations in the elaboration of both aversive and rewarding stimuli characterize several psychopathologies including anxiety, depression and addiction. Several studies suggest that different neurotrasmitters, within the corticolimbic system, are critically involved in the processing of positive and negative stimuli. Individual differences in this system, depending on genotype, have been shown to act as a liability factor for different psychopathologies. Inbred mouse strains are commonly used in preclinical studies of normal and pathological behaviors. In particular, C57BL/6J (C57) and DBA/2J (DBA) strains have permitted to disclose the impact of different genetic backgrounds over the corticolimbic system functions. Here, we summarize the main findings collected over the years in our laboratory, showing how the genetic background plays a critical role in modulating amminergic and GABAergic neurotransmission in prefrontal-accumbal-amygdala system response to different rewarding and aversive experiences, as well as to stress response. Finally, we propose a top-down model for the response to rewarding and aversive stimuli in which amminergic transmission in prefrontal cortex (PFC) controls accumbal and amygdala neurotransmitter response.

## Introduction

Adaptive behavior involves the ability to represent the value of positive or negative stimuli, establish predictions about them, and use these predictions to guide behavior (O’Doherty, 2004). Animals and humans have a propensity to seek out rewards, avoid punishments, and cope with negative situations, such as stressful events. Aberrations in the elaboration of aversive and rewarding stimuli characterize several psychopathologies, including anxiety, depression, and addiction. For instance, disorders of mood and motivation are frequently associated with anhedonia (reduced ability to experience pleasure), and alterations in neural processing of rewarding and aversive stimuli have been recently proposed as an endophenotype of depression (Hasler et al., 2004; McCabe et al., 2009; Ventura et al., 2013). Thus, understanding the neural mechanisms by which positive and aversive stimuli are elaborated is critical for the development of therapeutic approaches for several psychopathologies.

Mood and motivation disorders and other psychiatric conditions have complicated etiologies and result from complex interactions between genetic and environmental precipitating factors. In psychobiology studies, inbred strains are a useful tool to investigate the role of genetic factors, in interaction with aversive and rewarding experiences, in susceptibility to development and expression of psychopathology. In particular, data from C57 and DBA have provided information on how the response of specific neuronal systems is related to genetic background.

### The use of the inbred strains

The use of inbred strains of mice offers great advantages to studies aimed to determine the function of neurotransmitter systems with regard to the effects of psychotropic drugs, stressful events, and various psychopathologies.

An inbred strain is a set of animals that is produced by at least 20 consecutive generations of sister-brother or parent-offspring matings and that can be traced to a single ancestral pair in the 20th or subsequent generations. Inbred animals are nearly entirely homozygous, providing a well-defined and consistent genotype for analysis. The genetic stability of inbred strains over the years and through laboratories has allowed myriad relevant information for several commonly used strains to be accumulated. Thus, comparative studies on neurotransmitter activity in various regions of the brain in inbred mouse strains, which have differences of behavioral outcomes, is one approach to investigate the neurochemical bases of behavioral expression. In any experimental procedure that involves laboratory-bred stocks, the results might reflect the strain and species that are used. There is a significant amount of data on differences in the effects of various experimental conditions against which the findings from inbred strains can be referenced, controlling the influence of this source of variability. Moreover, behavioral, pharmacological, physiological, and biochemical comparisons between inbred strains constitute a preliminary stage for more extensive genetic research, such as quantitative trait loci (QTL) analysis, to identify and map genes in mice. Such a strategy can facilitate extrapolation of the results to the human genome, due to the significant extent of the linkage homology between human and mouse (Plomin et al., 1991; Crabbe et al., 1994).

### C57BL/6 and DBA/2 strains

C57BL/6 (C57) and DBA/2 (DBA) mice are among the most frequently studied inbred strains with regard to psychobiology because their behavioral responses have strain-dependent differences. Moreover, the functional and anatomical characteristics of their brain neurotransmitter systems have been examined extensively in these strains. A wealth of data on various parameters, such as neurotransmitter metabolism and release, receptor density and distribution, and activity of second messengers, has been accumulated. Consequently, data collected in these strains can offer important indications about the relationship between the behavioral and central effects of different neurotransmitters and, more generally, the involvement of brain neurotransmitters in the control of behavior.

Clinical and preclinical studies suggest that the prefrontal cortex (PFC), striatum (including the nucleus accumbens (NAc)) and amygdala are activated by natural positive or negative salient stimuli, constituting a common substrate for processing rewarding and aversive stimuli (Berridge and Robinson, 1998; Becerra et al., 2001; Jensen et al., 2003; O’Doherty, 2004; Borsook et al., 2007). Aminergic and amino acid transmission are the principal modulatory mechanisms of the corticolimbic system, and the dysregulation of these systems is linked to alterations in the elaboration of aversive and rewarding stimuli underlying various psychopathologies.

The main findings collected over the years in our lab, by microdialysis experiments, have identified the role of several prefrontal cortex-accumbal-amygdala neurotransmitter systems in the elaboration of rewarding or aversive stimuli. Here we report findings from two experimental paradigms: place conditioning and forced swimming test (FST). The place conditioning paradigm permits to investigate the motivational salience attribution process to conditioned stimuli that are associated with primary rewarding and aversive events (Tzschentke, 1998; Reynolds and Berridge, 2002). The FST is one of paradigms most widely used to measure antidepressant activity of new drugs. Moreover, FST allows to assess alterations in depression-like behavior and coping response to stress in both normal and genetically modified animals (Porsolt et al., 1977; Borsini and Meli, 1988). The behavioral responses in the FST are thought to engage a coping strategy (Thierry et al., 1984), in which immobility behavior is an index of higher perceived motivational impact of a stressful experience. Finally, we used restraint stress to evaluate the time-dependent response induced by stress on different neurotransmitters in specific brain areas by intracerebral *in vivo* microdialysis.

## Biological basis of processing rewarding/aversive stimuli: prefrontal-amygdala-accumbal system

The NAc, together with the PFC and amygdala, can be considered a component of the brain network that regulates effort-related functions (Salamone and Correa, 2012). The prefrontal-accumbal cathecolamine and system has been demonstrated to play a critical role in processing both rewarding and aversive stimuli (Ventura et al., 2007). Moreover, the amygdala is involved in Pavlovian conditioning of emotional responses and modulates memory for arousing experiences (Balleine, 2005; Balleine and Killcross, 2006; McGaugh, 2006), and a complex anatomical and functional connection between the amygdala, medial prefrontal cortex (mpFC), and NAc has been reported (Del Arco and Mora, 2009 for review). Studies showed a crucial role of mpFC/amygdala system in both processing of rewarding and coping to aversive stimuli, including stress conditions (Robinson and Berridge, 1993; Becerra et al., 2001; Gottfried et al., 2002; Jensen et al., 2003; Borsook et al., 2007; Andolina et al., 2013; Rudebeck et al., 2013). A growing body of evidence indicates that the prefrontal aminergic system controls both dopamine (DA) release in the NAc and GABA release in the amygdala, sub-cortical areas that mediate the elaboration of rewarding and aversive stimuli. Moreover, an alteration of this process seems to characterize several psychopathologies, including anxiety, depression, and addiction. Twin and adoption studies have demonstrated a gene-environment interaction in the development of psychiatric disorders, indicating that genetic background modulates the capacity of an environmental risk factor to give rise to mental illness (Caspi and Moffitt, 2006). In preclinical studies, inbred strains are a useful tool to investigate the role of genetic factors, in interaction with aversive and rewarding experiences, in susceptibility to development and expression of psychopathology. Indeed, comparative study of brain neurotransmitter activity and behavior in different genetic backgrounds is a major strategy for determining the neural basis of rewarding and aversive effects in relation to individual differences. In particular, the C57 and DBA strains have allowed us to determine the impact of genetic background on corticolimbic system function.

### Rewarding stimuli

The principal function of DA in motivational salience processes and in the elaboration of rewarding stimuli has been widely reported (Robinson and Berridge, 2001). Thus, increased DA transmission in the NAc mediates the rewarding/reinforcing effects of addictive drugs (Di Chiara and Imperato, 1988; Wise and Rompre, 1989; Pontieri et al., 1995; Koob et al., 1998; Robbins and Everitt, 1999; Ventura et al., 2003, 2005, 2007). However, recent evidence suggests major involvement of brain norepinephrine (NE) in the behavioral and central effects of rewarding pharmacological and natural stimuli (Darracq et al., 1998; Tassin, 1998; Drouin et al., 2001; Zarrindast et al., 2002; Ventura et al., 2007; Latagliata et al., 2010; Puglisi-Allegra and Ventura, 2012). Ventura et al. demonstrated that selective prefrontal NE depletion in mice abolished the increase of DA in the NAc induced by various classes of drugs of abuse and food (Ventura et al., 2003, 2005, 2007, 2008; Latagliata et al., 2010). Moreover, these studies reported that an intact prefrontal cortical NE is necessary for Conditioned Place Preference (CPP) induced by amphetamine, morphine, cocaine, ethanol, and chocolate as well as for reinstatement (relapse) of extinguished morphine-induced CPP and for ethanol intake in a choice test (Ventura et al., 2003, 2005, 2006, 2007; Latagliata et al., 2010). Thus, they demonstrate that prefrontal NE transmission is crucial for accumbal DA release induced by pharmacological and natural rewarding stimuli and processing the rewarding/reinforcing effects of these stimuli. Finally, in addition to the prefrontal noradrenergic/accumbal dopaminergic neuronal circuit, different studies showed a crucial role of mpFC/amygdala system in processing of rewarding stimuli (Robinson and Berridge, 1993; Becerra et al., 2001; Gottfried et al., 2002; Borsook et al., 2007; Rudebeck et al., 2013).

Inter-individual differences have been frequently reported in the elaboration of both rewarding and aversive stimuli. Genotype-dependent control of corticolimbic neurotransmission could be responsible for individual differences in the elaboration of positive and aversive stimuli and thus be linked to different susceptibility to psychopathologies. Our findings in C57 and DBA mice support this hypothesis. Concerning the elaboration of rewarding stimuli, we showed differential effects of drugs of abuse (amphetamine), depending on genotype (Table 1). For instance, mice of DBA background are hyporesponsive to the behavioral effects of D-amphetamine, whereas C57 mice are highly responsive to the stimulating/reinforcing effects of amphetamine, as evidenced by increased locomotor activity, and amphetamine-induced CPP (Zocchi et al., 1998; Cabib et al., 2000; Ventura et al., 2004). Amphetamine produces lower prefrontal and higher accumbal DA levels, as well as higher locomotor activity, in C57 mice in comparison with DBA mice (Ventura et al., 2004). Moreover, selective prefrontal DA depletion in DBA mice leads to high DA outflow in the NAc and hyperlocomotion, comparable to those observed in C57 mice (Ventura et al., 2004). This evidence demonstrates that mesocortical DA controls the genotype-dependent effects of systemic amphetamine on mesoaccumbens DA release and locomotion. Nevertheless, as we have stressed, noradrenergic transmission in the mpFC has significant modulatory function on accumbal dopaminergic transmission and mediates the rewarding/reinforcing effects of addictive drugs. Because prefrontal DA inhibits NAc DA, whereas NE has been suggested to be enabling (Darracq et al., 1998), we hypothesized that an imbalance in NE/DA in the mpFC regulates DA in the NAc and the related behavioral outcomes, rendering the C57 strain more responsive than DBA. This hypothesis was confirmed by experiments that demonstrated that selective prefrontal cortical NE depletion abolishes the effects of amphetamine on DA in the accumbens and CPP in C57 mice (Ventura et al., 2003), whereas selective prefrontal DA depletion (sparing NE) leads to DA outflow in the NAc and behavioral outcomes in DBA mice, similar to those of C57 (Ventura et al., 2004, 2005). Thus, these evidences demonstrated that genotype-dependent susceptibility to the addictive properties of drugs of abuse (amphetamine) involves imbalanced DA and NE transmission in the mesocorticolimbic system.

**Table 1:** **Summary of behavioral response and neurotrasmitter release to rewarding and aversive stimuli shown by C57 and DBA mice**.

	Rewarding stimuli (amphetamine)				References
	Behavior		Neurotrasmitter release		
	CPP	Loc Act	mpFC	NAc	
C57	↑				Cabib et al. (2000)
DBA	↓				Ventura et al. (2003)
C57		↑	DA ↓	DA ↑	Ventura et al. (2003, 2004)
DBA		↓	DA ↑	DA ↓	Zocchi et al. (1998)
	**Aversive stimuli (stress)**				
	**Behavior**	**Neurotrasmitter release**			
	**Immobility (FST)**	**mpFC**	**NAc**	**Amy**	
C57	↑	5-HT ↑ (restraint)		GABA ↑ (restraint)	Andolina et al. (in press)
DBA	↓	5-HT ↓ (restraint)		GABA ↓ (restraint)	
C57	↑	DA ↑ (FST/restraint)	DA ↓ (FST/restraint)		Ventura et al. (2001, 2002)
DBA	↓	DA ↓ (FST/restraint)	DA ↑ (FST/restraint)	

*↑ increase or ↓ decrease in comparison with DBA. Abbrevation: CPP, Conditioned Place Preference; Loc Act, Locomotor activity; FST, Forced swimming test; mpFC, medial prefrontal cortex; NAc, nucleus accumbens; Amy, Amygdala; DA, Dopamine; 5-HT, Serotonin*.

### Aversive stimuli

Aversive pharmacological and natural experiences, such as lithium and stress administration, have been shown to activate the same prefrontal cortical-subcortical network affected by rewarding stimuli (Pascucci et al., 2007; Ventura et al., 2007, 2008). In fact, the authors observed that natural and pharmacological aversive stimuli induce a clear-cut increase of NE in the mpFC and DA in the NAcs that was abolished by selective prefrontal NE depletion (Ventura et al., 2007, 2008). Results concerning natural, non-pharmacological aversive experiences (restraint stress) have demonstrated the same prefrontal noradrenergic control on accumbal DA outflow in rats (Pascucci et al., 2007). This study showed that exposure to a novel stressor (restraint) promotes a rapid, massive, and transient increase in NE release in the mpFC, paralleling the rise in mesoaccumbens DA release (Pascucci et al., 2007). Selective prefrontal NE depletion prevents both the cortical NE response and the increase in accumbens DA release, thus confirming the modulatory function of prefrontal NE transmission in accumbal DA transmission induced also by aversive stimuli. All together, these data demonstrate that catecholaminergic transmission in the neural circuit comprising NAc and mpFC is crucial in both stress response and processing of negative and positive stimuli (Cabib et al., 1988; Le Moal and Simon, 1991; Pascucci et al., 2007; Ventura et al., 2007, 2013; Cabib and Puglisi-Allegra, 2012).

In addition to the mpFC noradrenergic/accumbal dopaminergic neuronal circuit, other brain areas and neurotransmitters, such amygdala GABAergic transmission, are likely to be engaged in these processes. Concerning the prefrontal-amygdala system, we have recently showed that amygdalar GABA regulation by prefrontal 5-HT is critical for processing stressful experiences and for determining passive coping outcomes, as measured by FST in mice (Andolina et al., 2013). We have demonstrated that a stressful experience, such as restraint, increases 5-HT levels in the mpFC and GABA levels in the amygdala and that selective depletion of cortical 5-HT canceled out these stress-induced responses, implicating prefrontal 5-HT in the control of GABAergic transmission in the amygdala during stress exposure. Sustained stress-induced 5-HT outflow in the mpFC and GABA outflow in the basolateral amygdala (BLA) lead to sustained immobility. However, a disconnection between prefrontal 5-HT and amygdalar (BLA) GABAergic transmission leads to low immobility in the FST (Andolina et al., 2013). These results highlight other critical neural mechanisms in the perceived motivational impact of stressful experiences in which the prefrontal/amygdala connectivity mediated by 5-HT and GABA transmission has a significant function. Concerning data from inbred strains of mice, our restraint and FST results in C57 and DBA mice showed a genotype control of corticolimbic neurotransmission (Table 1). We found that restraint stress inhibited mesoaccumbens DA release, which was accompanied by rapid and strong activation of mesocortical DA metabolism in C57 mice; the opposite pattern occurred in DBA mice, thus demonstrating genetic control over the balance between mesocortical and mesoaccumbens DA responses to stress (Ventura et al., 2001). Moreover, C57 but not DBA mice experienced high immobility in their first session of the FST and immediate and robust activation of mesocortical DA metabolism and inhibition of mesoaccumbens DA metabolism and release. In addition, the behavioral and mesoaccumbens DA responses to FST in C57 mice were reduced and reversed, respectively, by selective dopamine DA depletion in the mpFC (Ventura et al., 2002). These studies showed that, as with rewarding stimuli, the genetic background governs the susceptibility to stressful experiences through the mesocortical-limbic DA response.

Another important neural network mediating stress responses is the mpFC-amygdala circuit that has been shown to be influenced by genotype (Holmes, 2008 for review). Specifically, consistent with the evidence that we have discussed, C57 mice have been reported to display greater immobility in the FST compared to DBA mice (Alcaro et al., 2002; Ventura et al., 2002). DBA and C57 mice are characterized by a different prefrontal 5-HT (Calcagno et al., 2007; Andolina et al., in press). In particular, DBA mice present lower 5-HT transporter binding and lower immobility in the FST than C57 (Sugimoto et al., 2008; Popova et al., 2009). Moreover, DBA mice are homozygous for the 1473G allele TPH-2, linked to low 5-HT synthesis rate, while C57BL/6 mice are homozygous for the 1473C allele. This allelic variant in DBA causes lower brain 5-HT synthesis than in C57BL/6 mice carrying the “C” allele (Zhang et al., 2004; Cervo et al., 2005). Moreover, differences between C57 and DBA mice have been reported for amygdala functioning which have been linked to strain-dependent difference in stress responsiveness (DuBois et al., 2006; Yang et al., 2008; Mozhui et al., 2010). Consistent with the evidences that genetic variation in cortico-amygdala system contributes to individual differences in stress response and to stress-related behavior, recently we reported that C57 mice show higher 5-HT outflow in the mpFC and higher GABA outflow in the BLA induced by stress (restraint) compared with the DBA strain. (Andolina et al., in press).

All together, these data indicate that strain-dependent prefrontal corticolimbic regulation, probably through different neurotransmitter systems including NE, DA, 5-HT, and GABA determines the differences in stress-coping behaviors in the FST in C57 and DBA mice.

## Conclusion

The evidences that we have discussed demonstrate that neurotransmission in the neural circuit comprising PFC, NAcs, and amygdala, is crucial for processing rewarding and aversive stimuli. Moreover, data on C57 and DBA strains demonstrate how the genetic background determines the intensity and effects of the response to positive and negative stimuli. Most of the experiments that we have discussed above describe how selective NE, DA, or 5-HT depletion in mpFC modifies the neurotransmitter response of subcortical structures, such as the NAc and amygdala. PFC sends glutamatergic outputs to subcortical areas, including the NAc and amygdala, that mediate motor, emotional, and mnemonic function. In a top-down model, alterations in PFC neurotransmission could modify the function of specific PFC cellular networks (Yang and Chen, 2005; Del Arco and Mora, 2009) and, consequently, the function of subcortical structures, including the NAc and amygdala. This could lead to the development of abnormal behaviors associated with psychiatric disorders, such as depression, anxiety, and addiction. Our data support this model, wherein the aminergic system in the mpFC has a central role in dopaminergic and GABAergic neurotransmission in the NAc and amygdala, respectively. Furthermore, our findings support a model of genotype-dependent control of the prefrontal-accumbal-amygdala neural circuit, which could mediate the differential behavioral responses to many natural and pharmacological rewarding and aversive stimuli.

## Conflict of interest statement

The Review Editor Antonella Gasbarri declares that, despite being affiliated to the same institution as author Diego Andolina, the review process was handled objectively and no conflict of interest exists. The authors declare that the research was conducted in the absence of any commercial or financial relationships that could be construed as a potential conflict of interest.
